# Supplementary figures and images for: Traditional surveys versus ecological momentary assessments: Digital citizen science approaches to improve ethical physical activity surveillance among youth
Source: PLOS Digit Health. 2023 Sep 27;2(9):e0000294. doi: 10.1371/journal.pdig.0000294 (PMC10529555; doi:10.1371/journal.pdig.0000294)

# Supporting Information

**S1 Fig: Study design diagram**

**
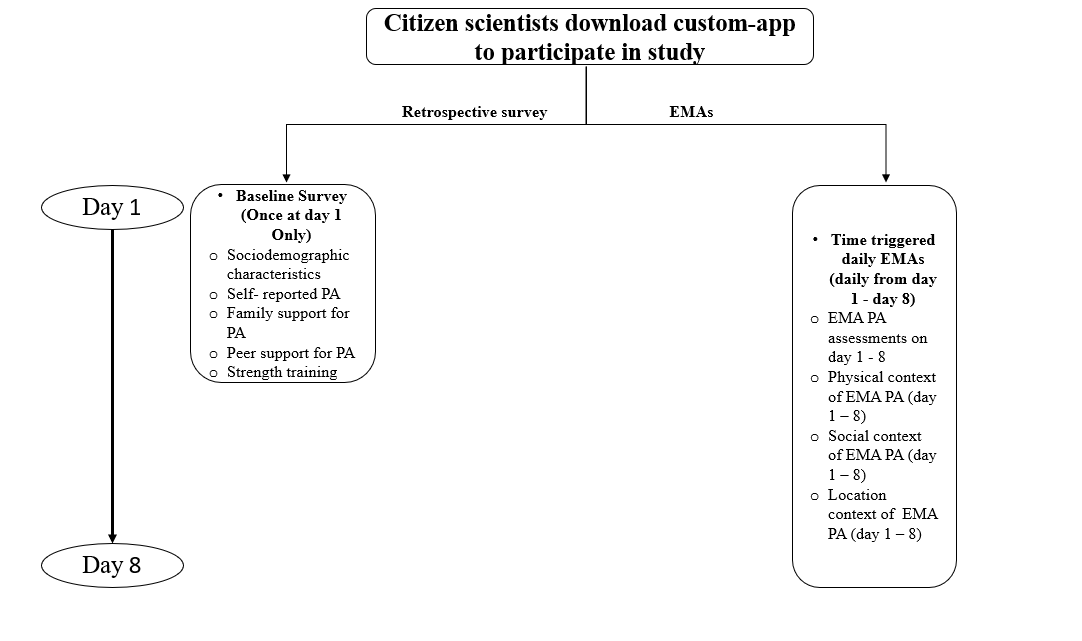
**

Supplement: S1 Fig — (DOCX) [file pdig.0000294.s001.docx]

**S2 Fig: Digitally deployed modified retrospective PA survey.**


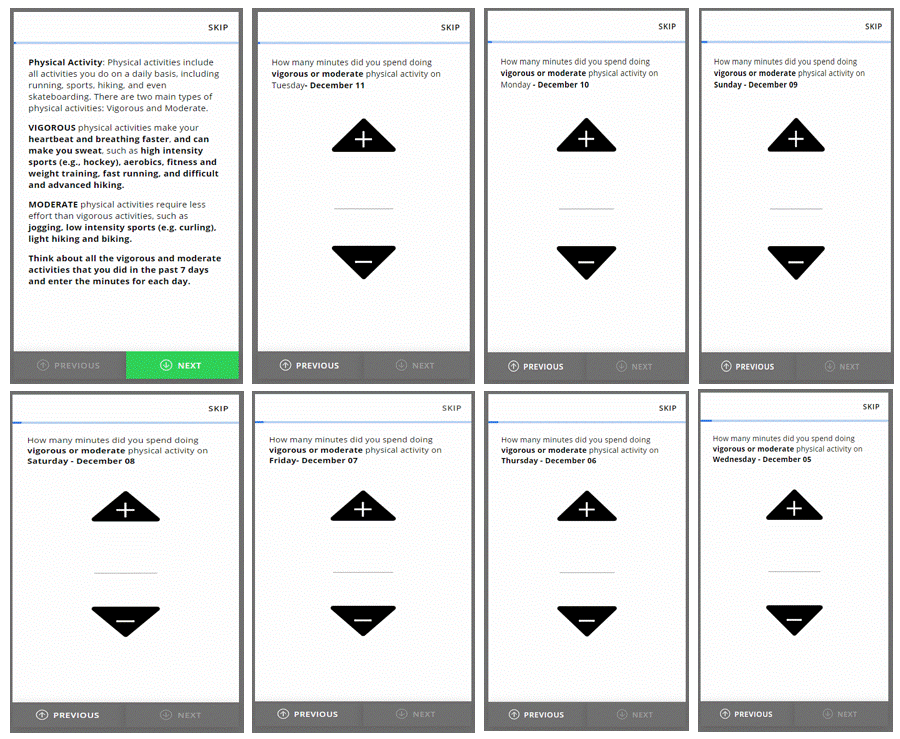

Supplement: S2 Fig — (DOCX) [file pdig.0000294.s002.docx]

**S3 Fig: EMAs PA duration.**


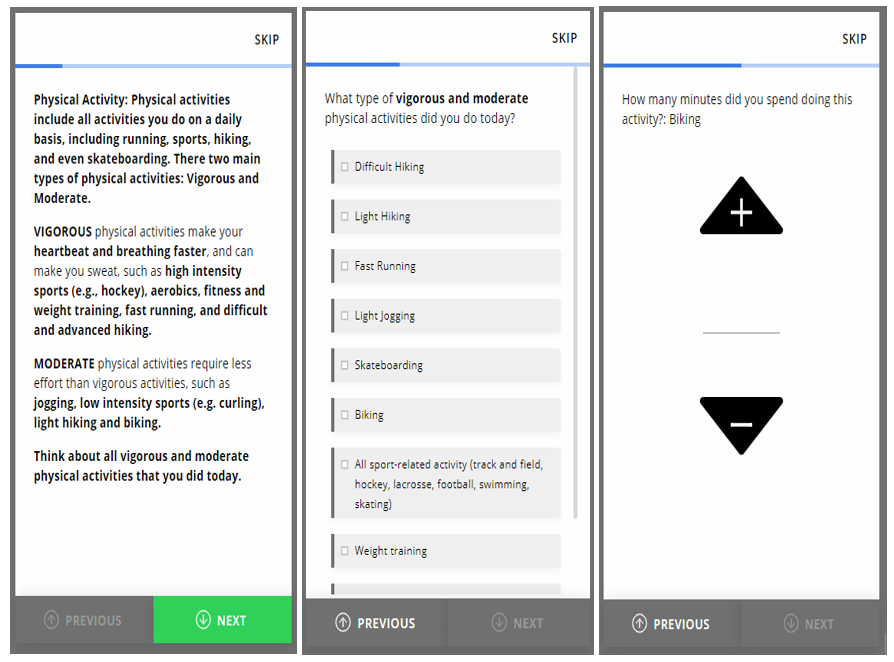

Supplement: S3 Fig — (DOCX) [file pdig.0000294.s003.docx]
